# The Influence of Cancer Stem Cells on the Risk of Relapse in Adenocarcinoma and Squamous Cell Carcinoma of the Lung: A Prospective Cohort Study

**DOI:** 10.1093/stcltm/szab029

**Published:** 2022-02-23

**Authors:** Valentina Masciale, Federico Banchelli, Giulia Grisendi, Roberto D’Amico, Antonino Maiorana, Alessandro Stefani, Uliano Morandi, Franco Stella, Massimo Dominici, Beatrice Aramini

**Affiliations:** 1 Division of Thoracic Surgery, Department of Medical and Surgical Sciences, University of Modena and Reggio Emilia, Modena, Italy; 2 Division of Oncology, Department of Medical and Surgical Sciences, University of Modena and Reggio Emilia, Modena, Italy; 3 Center of Medical Statistic, Department of Medical and Surgical Sciences, University of Modena and Reggio Emilia, Modena, Italy; 4 Institute of Pathology, Department of Medical and Surgical Sciences, University of Modena and Reggio Emilia, Modena, Italy; 5 Division of Thoracic Surgery, Department of Experimental, Diagnostic and Specialty Medicine—DIMES of the Alma Mater Studiorum, University of Bologna, G.B. Morgagni—L. Pierantoni Hospital, Forlì, Italy

**Keywords:** cancer stem cells, early stages, locally advanced stage, non–small cell lung cancer, relapse

## Abstract

**Purpose:**

Lung cancer relapse may be associated with the presence of a small population of cancer stem cells (CSCs) with unlimited proliferative potential. Our study assessed the relationship between CSCs and the relapse rate in patients harboring adenocarcinoma (ADL) and squamous cell carcinoma of the lung (SCCL).

**Experimental design:**

This is an observational prospective cohort study (NCT04634630) assessing the influence of CSC frequency on relapse rate after major lung resection in 35 patients harboring early (I-II) (*n* = 21) and locally advanced (IIIA) (*n* = 14) ADL and SCCL. There was a 2-year enrollment period followed by a 1-year follow-up period. Surgical tumor specimens were processed, and CSCs were quantified by cytofluorimetric analysis.

**Results:**

Cancer stem cells were expressed in all patients with a median of 3.1% of the primary cell culture. Primary analysis showed no influence of CSC frequency on the risk of relapse (hazard ratio [HR] = 1.05, 95% confidence interval [CI] = 0.85-1.30). At secondary analysis, patients with locally advanced disease with higher CSC frequency had an increased risk of relapse (HR = 1.26, 95% CI = 1.14-1.39), whereas this was not observed in early-stage patients (HR = 0.90, 95% CI = 0.65-1.25).

**Conclusion:**

No association was found between CSC and relapse rates after major lung resection in patients harboring ACL and SCCL. However, in locally advanced-stage patients, a positive correlation was observed between CSC frequency and risk of relapse. These results indicate a need for further molecular investigations into the prognostic role of CSCs at different lung cancer stages.

**Clinical Trial Registration:**

NCT04634630.

## Graphical Abstract

**Graphical Abstract:**
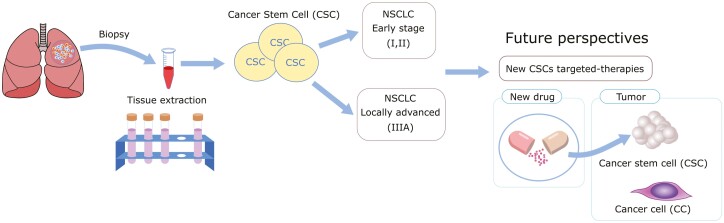

**Lessons Learned**

• In lung cancer, long-term survival after surgery remains quite low.

•  Postoperative recurrence of non-small cell lung cancer occurs in the first 5 years, in 20% to 75% of patients.

•  The identification of driver mutations has induced the development of new targeted treatments, although many patients still do not respond to them.

•  Cancer stem cells (CSCs) have been shown to have capacities of normal stem cells, such as self-renewal, high clonogenic potential, unlimited growth, tumor-initiating capacity, and drug resistance.

•  There is an urgent need to investigate the prognostic role of CSCs at different lung cancer stages to set future stage-tailored therapies.

**Significance Statement**

Stratifying patients by stage shows a positive correlation between cancer stem cell frequency and time to relapse for locally advanced lung cancer.

## Introduction

A major issue with solid tumors is the frequency of relapse, especially in advanced diseases. The risk of recurrence is much higher in advanced cancer patients; it also heavily depends on the tumor type.^1-4^ In lung cancer, surgical treatment offers the best prognosis in patients with primary non–small cell lung cancer (NSCLC),^5^ even though long-term survival after surgery remains quite low.^6^ Postoperative NSCLC recurrence occurs in the first 5 years, ranging from 20% to 75% of patients. Most recurrences (>80%) take place in the first 2 years after surgery.^6-12^

This aspect is of particular importance in the case of advanced diseases affecting the lung since most patients die from a recurrence.^13^ So far, the different recurrence rates have no definitive explanation in oncology. For decades, the scientific community has sought to determine the role of recurrence and to identify possible risk factors for cancer progression and resistance to therapy, such as hereditary or somatic mutations in solid organs,^14,15^ phenotypic characteristics, environmental characteristics, and habits of individual patients.^16^ Recently, a genetic susceptibility has been identified that contributes to approximately 8% of lung cancer through genetic mutations that enhance the risk of the disease progression.^13,17^ The discovery of driver genes for NSCLC marked a breakthrough in its treatment, helping oncologists to choose optimal therapies.^18,19^ In particular, the identification of the driver mutations in NSCLC over the last 10 years has improved the understanding of lung cancer pathogenesis and has also led to the use of specific targeted therapies. Nevertheless, there remain patients who do not respond to these treatments.^20^ Resistance to therapy can occur primarily (that is *de novo*) or may develop after exposure to targeted agents, and it can exist as resistant clones within a tumor or in different tumors within the same patient.^21^ In addition to the targeted therapy, some patients are treated with immunotherapy (IT) designed to stimulate a patient’s own immune system to recognize and then eliminate cancer cells.^22-25^ Lung cancer immunotherapies are mainly focused on programmed death ligand 1 (PD-L1) and its T-cell receptor (PD-1), and in recent years they have improved survival in patients with early to advanced NSCLC when used in combination with chemotherapy in advanced stages.^25-28^ However, some patients still do not respond to IT, and this aspect represents a huge issue in metastatic—and, more recently, early adjuvant—settings.^29^ This lack of response may be due to NSCLC plasticity, which is also linked to a small population of cancer stem cells (CSCs) that have unlimited proliferative potential and self-renewal ability.^30,31^ Cancer stem cells were first isolated from original tumor tissue using CD34 and CD38 surface marker expression,^32,33^ but, in the last decade, aldehyde dehydrogenase (ALDH) has become the most commonly used method to select CSCs from solid tumors.^34-36^ In particular, ALDH is an enzyme responsible for the oxidation of aldehydes to carboxylic acids which is considered important for detoxing several aldehydes, the synthesis of retinoic acid (RA), and other biological regulators involving the cellular functions.^35^ During the last 10 years, ALDH has been studied as a selectable marker for identifying the tumor-initiating stem-like cells,^37-41^ and also for the most aggressive CSCs.^42, 43^ Cancer stem cells, first described in leukemia,^32^ were subsequently identified in breast cancer^44^ and other solid tumors with many of the capacities of normal stem cells, such as self-renewal, high clonogenic potential, unlimited growth, tumor-initiating capacity, and drug resistance. Due to these characteristics, CSCs have proven invincible to date, mainly because they are indestructible due to their lack of identifying markers. Jiang et al demonstrated in 2009 that CSC derived from NSCLC contains a population of the ALDH1-positive cells that may generate tumors recapitulating the heterogeneity of the parental cancer cells.^45^ In 2010 Sullivan et al^46^ gained further insights regarding ALDH in 45 NSCLC cell lines and in 11 NSCLC patient tumors, confirming that these ALDH-positive to ALDH^high^ cells were associated with the NOTCH signaling pathway and with the well-known characteristics of CSCs.^47^ For these reasons, ALDH expression has been used for the identification of CSCs in many solid tumors. However, no studies are correlating ALDH with recurrence, even though CSCs have a key role in the diagnosis and treatment of tumors because they are the main cause of tumor relapse and resistance to common oncological treatments. In addition, ALDH has been used as a prognostic marker in many cancer types, including pancreatic cancer: immunohistochemical analysis of 97 patients with pancreatic cancer revealed an association of ALDH1A with poor survival.^48-50^ More recently, a meta-analysis of 1926 patients revealed a high correlation of the stem cell marker ALDH1 with tumor TNM staging and lymph node metastasis.^49^ However, no studies have correlated ALDH with recurrence, even though CSCs have a key role in the diagnosis and treatment of tumors, because they are the main cause of tumor relapse as well as tumors resistant to common oncological treatments.^50^ To that end, our prospective cohort study is the first attempt to evaluate the relationship between CSCs and disease-free survival in patients harboring adenocarcinoma and squamous cell carcinoma of the lung by sorting the CSCs.

## Methods

### Aim and Outcomes

The primary aim was to assess the influence of CSC frequency on disease-free survival in patients harboring NSCLC. The secondary aim was to assess this influence in the following 4 patient subgroups: stage I or II NSCLC (early stages); stage IIIA NSCLC (advanced stages); adenocarcinoma of the lung (ADL); and squamous cell carcinoma of the lung (SCCL). The outcome of the study was disease-free survival measured as the time from surgical tumor resection to relapse.

### Study Design

This was an observational prospective cohort study. The study, which involved human subjects, human material, and human data, was performed in accordance with the Declaration of Helsinki and was approved by the Ethics Committee at the University Hospital of Modena in March 2017. All patients signed an informed consent form before enrollment. The study was registered on ClinicalTrials.gov (NCT04634630) and was described following the STROBE guidelines.^51^ The study was planned to have a 2-year enrollment period followed by a 1-year follow-up period. This guaranteed that the individual follow-up time ranged from a minimum of 1 year to a maximum of 3 years.

### Enrollment

Patients who underwent major lung resection by video-assisted thoracoscopic surgery (VATS) or by lateral thoracotomy at the Division of Thoracic Surgery of Modena University Hospital (Italy) for stage I, II, or IIIA ADL or SCCL between September 2017 and September 2019 were eligible for inclusion. Inclusion criteria were as follows: age between 18 and 85 years; R0 resection; availability of formalin-fixed, paraffin-embedded surgery specimen from the primary tumor; and availability of fresh surgical specimen for cytofluorimetric analysis. Exclusion criteria were as follows: incomplete resection; unknown TNM status; synchronous tumors; and previous lung cancer.

### Cancer Stem Cells Frequency in Primary Tumor Cell Cells

The Aldefluor assay kit (Stemcell Technologies, Vancouver, CA) was used to identify and isolate cells with high ALDH enzymatic activity. Surgical tumor specimens were dissociated by tumor dissociation kit (Miltenyi Biotec, Italy) within 2 h of retrieval to obtain primary cell suspension for cell sorting and cytofluorimetric analysis using a BD FACSAria III (Becton Dickinson, Franklin Lakes, NJ). The gating strategy was described in Masciale et al.^52^ The CSC frequency was expressed as the percentage of ALDH^high^ cells among all viable cells. The fluorescent BODIPY-aminoacetate, produced by cells with ALDH enzyme activity, was quantified in the fluorescein isothiocyanate/FL1 channel (FITC), and only the brightest cells were quantified and sorted (<0.5%).

### Follow-up

All patients were followed up from lung resection until September 2020 or until recurrence, whichever came first. The time of cancer relapse was the time span between the recurrence diagnosis, based on clinical grounds, and the day of surgery. Both distant and local recurrences were considered. Patients who died before experiencing recurrence were considered to have censored follow-up times.

### Sample Size

We assumed that 50% of the patients would experience recurrence within a 2-year period. The recurrence rate was assumed based on the follow-up data gathered at the University Hospital of Modena. Based on these assumptions and on a target hazard ratio (HR) of 1.15 for every 1% increase in CSC frequency, and on a standard deviation of CSC frequency equal to 5%, a sample of approximately 32 patients guaranteed a 95% confidence level and 80% statistical power. This figure was rounded to 35 patients to account for participants who could be lost during follow-up. Concerning the secondary aim of subgroup analysis, we calculated that a sample of 19 patients would guarantee 95% confidence level and 80% statistical power if considering a target HR of 1.20. These sample size calculations were carried out by using Stata 13 statistical software (Stata Corp., College Station, TX) considering a Cox model with one regression slope.

### Statistical Analysis

The characteristics of patients and surgical specimens were described as mean ± standard deviation for quantitative variables or as absolute and percentage frequencies for categorical variables. Comparison of numeric variables between groups was assessed with the Wilcoxon-Mann-Whitney (WMW) test. The relapse rate was calculated as the number of events per 100 person-years and the median relapse-free time was assessed using the Kaplan-Meier method. Comparisons of Kaplan-Meier survival curves were assessed with the log-rank test. The association between the CSC frequency and the hazard of relapse was assessed using a Cox regression model with robust standard errors.^53^ The results were reported as HR associated with a 1% increase in CSC frequency. Both unadjusted and confounder-adjusted HRs were reported, considering sex (male vs. female), age (years), clinical stage (early vs. locally advanced), and tumor histotype (ADL vs. SCCL) as potential confounding variables. The primary analysis was carried out by analyzing the whole sample of NSCLC patients, whereas the secondary subgroup analyses were carried out by including interaction terms in the Cox model. The interactions of interest were CSC frequency × clinical stage (early stages or locally advanced stage) and CSC frequency × histotype (ADL or SCCL). Hazard ratios for the influence of CSC on relapse rates within these subgroups of patients were calculated as linear combinations of model parameters. The statistical analyses were carried out using R 3.6.3 software (The R Foundation for Statistical Computing, Wien) at the 95% confidence level (*P* < .05).

## Results

### Enrollment

**Figure 1.**
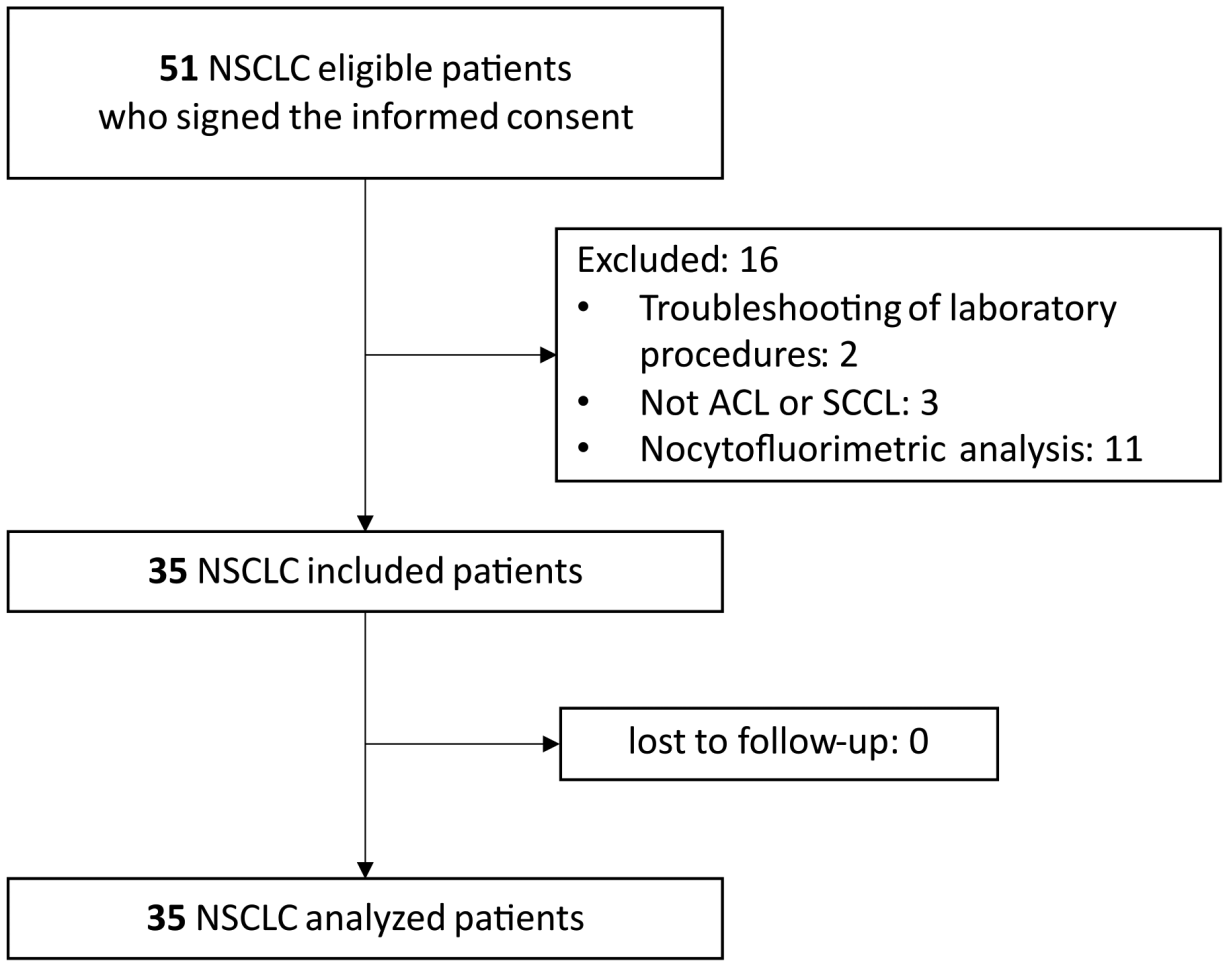
Flow chart of study enrolment.

From October 2017 to September 2019, 51 patients signed the informed consent and were eligible for inclusion in the study. Of these, 16 were excluded for the following reasons: 2 patients’ samples were used for troubleshooting of laboratory procedures; 3 patients were not harboring ADL or SCCL; and 11 patients had no fresh surgical specimen available for cytofluorimetric analysis. Thirty-five patients met the inclusion criteria and were followed up until September 2020. No patients were lost to follow up during the study (Fig. 1).

### Frequency of CSCs in NSCLC Patients

**Figure 2.**
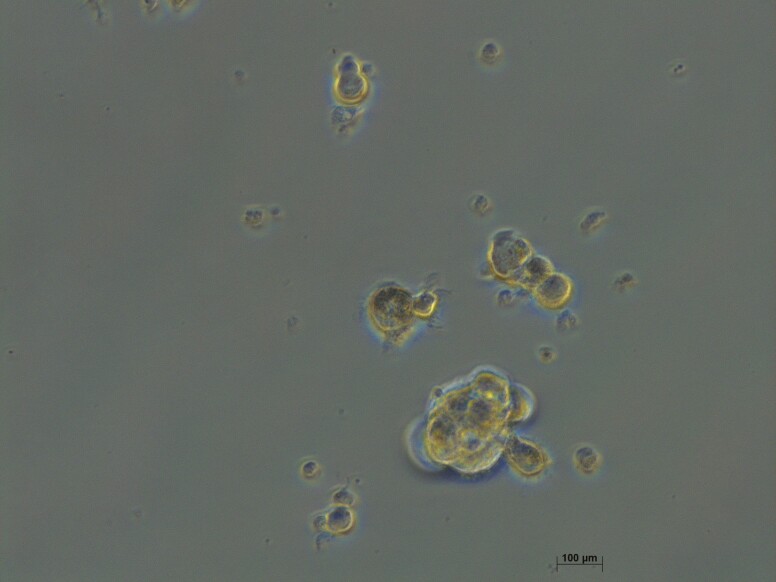
Lung tumorspheres. In vitro at 7 days of culture representative of the formation of lung tumorspheres of CSCs (ALDHhigh cells).

Characteristics of patients and surgical specimens are reported in Table 1. The average age was 70.5 ± 8.3 years, 65.7% of patients were male, and all were smokers. There were 21 (60.0%) patients with early-stage NSCLC and 14 (40.0%) with locally advanced NSCLC, and the ADL histotype was more frequent (74.3%) than SCCL (25.7%). Cancer stem cells were expressed in 35 (100.0%) patients’ surgical specimens, with a frequency ranging from 0.4% to 12.5% of all viable tumor cells. The average CSC frequency was equal to 3.6% ± 3.0%, whereas the median CSC frequency was 3.1% (interquartile range: 1.3% to 4.6%). The CSC frequency was similar between early and locally advanced-stage patients (4.1% ± 2.8% and 2.8% ± 3.1%, *P* = .127 by WMW test) as well as between patients harboring ADL or SCCL (3.4% ± 3.0% and 4.1% ± 2.9%, *P* = .446 by WMW test) (Fig. 2).

**Table 1.** Characteristics of patients

			All patients (*n* = 35)	Early-stage patients (*n* = 21)	Locally advanced-stage patients (*n* = 14)
Age	Years	mean ± SD	70.5 ± 8.3	72.6 ± 7.9	67.5 ± 8.1
		median (IQR)	70 (65-76)	72 (68-79)	68 (61-74)
Gender	Male	*n* (%)	23 (65.7)	12 (57.1)	11 (78.6)
Smoking habit	Current of former	*n* (%)	35 (100.0)	21 (100.0)	14 (100.0)
Pathological stage	Stage I	*n* (%)	11 (31.4)	11 (52.4)	-
	Stage II	*n* (%)	10 (28.6)	10 (47.6)	-
	Stage IIIA	*n* (%)	14 (40.0)	-	14 (100.0)
T	T1	*n* (%)	12 (34.3)	12 (57.1)	-
	T2	*n* (%)	9 (25.7)	9 (42.9)	-
	T3	*n* (%)	3 (8.6)	-	3 (21.4)
	T4	*n* (%)	11 (31.4)	-	11 (78.6)
N	N0	*n* (%)	26 (74.3)	16 (76.2)	10 (71.4)
	N1	*n* (%)	7 (20.0)	5 (23.8)	2 (14.3)
	N2	*n* (%)	2 (5.7)	0 (0.0)	2 (14.3)
M	M0	*n* (%)	35 (100.0)	21 (100.0)	14 (100.0)
Previous treatments	Neo adjuvant CT	*n* (%)	6 (17.1)	1 (4.8)	5 (35.7)
	Neo adjuvant RT	*n* (%)	0 (0.0)	0 (0.0)	0 (0.0)
	Adjuvant CT	*n* (%)	4 (19.0)	1 (4.8)	13 (92.9)
	Adjuvant RT	*n* (%)	0 (0.0)	0 (0.0)	0 (0.0)
Type of surgery	Lobectomy	*n* (%)	30 (85.7)	20 (95.2)	10 (71.4)
	Pneumonectomy	*n* (%)	5 (14.3)	1 (4.8)	4 (28.6)
Surgical approach	Lateral thoracotomy	*n* (%)	25 (71.4)	11 (52.4)	14 (100.0)
	VATS	*n* (%)	10 (28.6)	10 (47.6)	0 (0.0)
Diagnostic procedures	FGD-PET + FBS	*n* (%)	16 (45.7)	16 (76.2)	0 (0.0)
	FDG-PET + FBS + EBUS	*n* (%)	19 (54.3)	5 (23.8)	14 (100.0)
Histotype	ADL	*n* (%)	26 (74.3)	15 (71.4)	11 (78.6)
	Acinar	*n* (%)	13 (37.1)	9 (42.9)	4 (28.6)
	Papillary	*n* (%)	3 (8.6)	1 (4.8)	2 (14.3)
	Solid	*n* (%)	9 (25.7)	5 (23.8)	4 (28.6)
	Poorly differentiated	*n* (%)	1 (2.9)	0 (0.0)	1 (7.1)
	SCCL	*n* (%)	9 (25.7)	6 (28.6)	3 (21.4)
Pleural invasion	Yes	*n* (%)	18 (51.4)	7 (33.3)	11 (78.6)
Vascular invasion	Yes	*n* (%)	7 (20.0)	2 (9.5)	5 (35.7)
PET	SUVmax	mean ± SD	9.9 ± 6.6	7.6 ± 5.1	13.5 ± 7.2
		median (IQR)	8.4 (4.9-14.6)	6.2 (3.9-9.0)	13.7 (8.9-17.5)
Tumour dimension	mm	mean ± SD	51.1 ± 27.2	34.5 ± 15.2	75.9 ± 21.6
		median (IQR)	47 (29-69)	32 (25-40)	72 (63-80)
CSC frequency	% on viable cells	mean ± SD	3.6 ± 3.0%	4.1% ± 2.8%	2.8% ± 3.1%
		median (IQR)	3.1% (1.3-4.6%)	3.8% (1.8-5.4%)	2.0% (0.9-3.4%)

Abbreviations: SD, standard deviation; IQR, inter-quartile range; CT, chemotherapy; RT, radiotherapy; CSC, cancer stem cells; VATS, video thoracoscopic approach; FDG-PET, fluorodeoxyglucose-positron emission tomography; FBS, fibrobronchoscopy; EBUS, endobronchial ultrasound; ADL, adenocarcinoma of the lung; SCCL, squamous cell carcinoma of the lung.

### Disease-free Survival

The included patients were all followed up until September 2020 or until the occurrence of relapse, whichever came first. The total follow-up time was equal to 34.9 person-years, with an average follow-up equal to 364 days (range: 19-902 days). During this period, 26 patients (74.3%) experienced recurrence, with an incidence rate equal to 74.6 events per 100 person-years and with a median disease-free survival time equal to 0.94 years (95% confidence interval [CI] = 0.57-2.02). One patient (2.9%) died without experiencing recurrence. The disease-free probabilities in the whole sample at 1 and 2 years after surgery were equal to 47.5% (95% CI = 33.4-67.7%) and 29.4% (95% CI = 16.7-51.8%), respectively. Survival was related to clinical stage, as 14 patients harboring early-stage NSCLC experienced recurrence (66.7%) compared to 12 patients with locally advanced NSCLC (85.7%; *P* = .001 by log-rank test). The median disease-free survival times were 1.8 and 0.4 years, respectively. Conversely, no relevant difference was observed between the median disease-free survival times of ADL and SCCL, which were equal to 0.94 years and 0.86 years, respectively (*P* = .923 by log-rank test).

### Influence of CSC Frequency on Disease-free Survival

There was no influence of CSC frequency on disease-free survival in all stages of NSCLC (Table 2). In particular, a 1% increase in CSC frequency was not significantly associated with risk of relapse, in either the unadjusted analysis (HR = 0.99, 95% CI = 0.85; 1.16, *P* = .927) or in the adjusted analysis (HR = 1.05, 95% CI = 0.85; 1.30, *P* = .663). In the secondary subgroup analyses, we observed a differential effect of CSC frequency on disease-free survival among early and locally advanced stages of NSCLC (*P* = .038 for the interaction term). In particular, patients with higher CSC frequency had an increased risk of relapse in locally advanced NSCLC (HR = 1.26, 95% CI = 1.14; 1.39, *P* = .000 in the adjusted analysis), whereas this was not observed for early-stage NSCLC (HR = 0.90, 95% CI = 0.65; 1.25, *P* = .545 in the adjusted analysis). Conversely, no modification of effect was observed between the ADL and SCCL histotypes (*P* = .634 for the interaction term), and no relevant effect of CSC frequency on the risk of relapse was found within these 2 subgroups (Table 2).

**Table 2.** Influence of lung cancer stem cells frequency on disease-free survival.

Analysis	Unadjusted analysis			Adjusted analysis		
	HR	95% CI	*P*	HR	95% CI	*P*
**Primary analysis—all patients**						
All stages ADL and SSCL (*n* = 35)	0.99	0.85; 1.16	.927	1.05	0.85; 1.30	.663
**Secondary analysis—subgroups**						
Stage						
Early (I-II) (*n* = 21)	1.02	0.84; 1.22	.871	0.90	0.65; 1.25	.545
Locally advanced (IIIA) (*n* = 14)	1.23	1.06; 1.43	.007∗	1.26	1.14; 1.39	.000∗
Histotype						
ADL (*n* = 26)	0.99	0.82; 1.18	.875	1.02	0.82; 1.28	.830
SCCL (*n* = 9)	0.99	0.74; 1.32	.937	1.14	0.76; 1.73	.522

The table reports the average effect of a 1% increase in CSC frequency on disease-free survival.

Abbreviations: HR, hazard ratio; 95% CI, 95% confidence interval; *P*, *P*-value; ADL, adenocarcinoma of the lung; SCCL, squamous cell carcinoma of the lung.

∗Statistically significant at 95% confidence level (*P* < .05); The adjusted analysis considered sex (male or female), age (years), clinical stage (early or locally advanced) and histotype (ADL or SCCL) as the independent variables.

## Discussion

Despite having no clinical or pathologic evidence of residual disease, patients with completely resected node-negative NSCLC remain at risk for recurrence. The addition of novel biomarkers associated with poor survival^54,55^ has not improved the model’s ability to predict recurrence. In particular, several biomarkers, gene drivers, and molecules have been studied in the past decades with the focus on identifying a method capable of predicting recurrence and to better stratify lung cancer patients.^56-58^ In 2015, Raphael Bueno, in collaboration with Myriad Genetics, defined a prognostic score signature, cell-cycle prognostic (CCP) through a selection of 31 genes related to recurrence and defined in early-stage NSCLC-resected patients.^59^ However, at present, there is no common marker or prognostic approach able to better cure NSCLC in advanced stages or predict relapse for early stages.

Steps forward have been achieved with the knowledge gained in the field of CSCs as these cells are extremely important due to their capacity to self-renew, drive tumor formation and resist common medical oncological treatments.^55^ Cancer stem cells are responsible for the poor outcomes in many solid tumors, including lung cancer. However, despite the enormous amount of scientific literature describing CSCs in solid tumors, there is not sufficient evidence for a correlation between CSCs and lung cancer relapse.^49^ Thus, CSC detection has emerged as an extremely important challenge, particularly in the discovery of new therapeutic targets.^60,61^. This issue was recently described in a systematic review and meta-analysis by Dong Wei,^49^ in which the role of ALDH1 was investigated as a predictor of prognosis for cancer patients in 1926 cases, mostly with immunohistochemistry (IHC) and some cases with real-time PCR (RT-PCR) and immunofluorescence (IF). This meta-analysis showed that ALDH1 expression in lung cancer was correlated with a decrease in overall and disease-free survival. In this context, in which ALDH represents a marker of worst prognosis, our study aimed to investigate ALDH in lung cancer, directly studying its expression in primary cells extracted from surgical tumor specimens. In particular, we focused on quantifying the frequency of CSCs within the tumors of each patient by cytofluorimetric analysis, as a percentage of ALDH^high^ cells, connecting it with the time to tumor relapse in both ADL and SCCL.

Concerning our primary aim, we did not observe any relevant correlation between CSC frequency and relapse, as CSC frequency was not a risk factor in either the unadjusted or the adjusted analyses when considering patients with all stages of ADL and SCCL. However, when analyzing this correlation within subgroups of the enrolled population, some relevant findings emerged. First, this correlation was comparable between ADL and SCCL, probably because these types of tumor derive from the same “classified tumor” as NSCLC. Second, when stratifying patients between early-stage and locally advanced ADL and SCCL, 2 opposite results were observed. In locally advanced patients, there was a positive correlation between CSC frequency and risk of tumor relapse. As a direct consequence, on average, each increase of 1% in the frequency of CSCs in locally advanced patients yielded a 26% increase in the risk of relapse, indicating that CSC frequency could represent a strong predictor variable for patient prognosis. So far, no other prognostic parameter in NSCLC patients accounts for CSCs, whereas similar studies exist only for other cancer types. In addition, no significant correlation emerged when early-stage patients were analyzed. Even though the median percentage of CSCs is around 3% in both early and locally advanced stages, CSCs seem to have an impact on recurrence-free survival only in locally advanced stages. It is difficult to interpret this observation at this point, given the lack of information about CSCs broadly, but we believe there are important steps and differences not yet investigated in CSCs at early vs locally advanced stages. This idea is strongly supported by our recently published article^62^ regarding the presence of overexpressed cell-cycle genes linked to recurrence in both early and locally advanced stages, which may suggest that CSCs are in a “state of quiescence” in the early stages but become activated in locally advanced disease. More studies need to be done in the future to confirm and develop this hypothesis.

It is also important to underscore the value of our study regarding the methodology of CSC isolation. Usually, prognostic studies involving CSCs are based only on immunohistochemistry of formalin-fixed paraffin-embedded tissue, not including an analysis of cells derived from fresh tissue. This first observational study showing a connection between CSCs and lung cancer recurrence relies on data gathered from the analysis of the frequency of CSCs, directly isolated through cell sorting of primary cell suspensions derived from surgical tumor specimens. We focused on the quantification of CSCs within the tumor of each patient, measuring their frequency by cytofluorimetric analysis as a percentage of ALDH^high^ cells and connecting it with the time to tumor relapse in both ADL and SCCL. If these findings are confirmed in larger cohorts of patients, a better CSC characterization might be considered in further studies. Further research may also be needed to identify specific superficial markers ^63^ for the synthesis of new drugs that are tailored to CSCs, more responsive to lung cancer, and more able to prevent recurrence.^64^

In addition, the study of molecular CSC characteristics will better define the pathways behind them, which would be relevant for the identification of a marker able to stratify risk in oncology patients as well as to set molecular targets to predict and possibly prevent a recurrence.

### Limitations

The study has some limitations that may have affected the findings. First, so far there is no consensus on the use of ALDH for CSC measurement. However, ALDH is the most commonly used marker for CSCs, because in various types of cancers, ALDH^high^ cells display many characteristics of stem cells, such as self-renewal, clonogenicity, tumor-initiating capacity, and drug resistance.^65^ A second limitation may be related to the observed recurrence rates, which were higher than those reported by other authors, although this issue can be reasonably explained by our moderate sample size. Additionally, the observational nature of the study and the moderate sample size are limitations themselves because selection bias and confounding bias cannot be definitively ruled out. Moreover, inclusion of both ADL and SCCL patients may have introduced a source of heterogeneity, even if no confounding or modification effect was observed in our data. Based on these limitations, the generalizability of our results remains uncertain and further research on this topic is needed.

## Data Availability

The datasets used and/or analyzed during the current study are available from the corresponding author on reasonable request.
